# In Vitro Effect of Epigallocatechin Gallate on Heme Synthesis Pathway and Protoporphyrin IX Production

**DOI:** 10.3390/ijms25168683

**Published:** 2024-08-09

**Authors:** Daniela León, María Elena Reyes, Helga Weber, Álvaro Gutiérrez, Claudio Tapia, Ramón Silva, Tamara Viscarra, Kurt Buchegger, Carmen Ili, Priscilla Brebi

**Affiliations:** 1 Laboratory of Integrative Biology (LIBi), Centro de Excelencia en Medicina Traslacional (CEMT), Scientific and Technological Bioresource Nucleus (BIOREN), Universidad de La Frontera, Temuco 4780000, Chile; danielaines.leon@ufrontera.cl (D.L.); a.gutierrez05@ufromail.cl (Á.G.); c.tapia09@ufromail.cl (C.T.); tamara.viscarra@ufrontera.cl (T.V.); 2 Millennium Institute of Immunology and Immunotherapy, Santiago 8320165, Chile; kurt.buchegger@ufrontera.cl; 3 BMRC, Biomedical Reasearch Consortium-Chile, Santiago 8320165, Chile; 4 Departamento de Ciencias Básicas, Facultad de Medicina, Universidad de La Frontera, Temuco 4780000, Chile; 5 Instituto de Ciencias Biomédicas, Facultad de Ciencias de la Salud, Universidad Autónoma de Chile, Temuco 4810101, Chile; maria.reyes@uautonoma.cl (M.E.R.); ramon.silva@uautonoma.cl (R.S.); 6 Biomedicine and Traslational Research Laboratory, Centro de Excelencia en Medicina Traslacional (CEMT), Universidad de La Frontera, Temuco 4780000, Chile; helga.weber@ufrontera.cl; 7 Doctorado en Ciencias Mención Biología Celular y Molecular Aplicada, Universidad de La Frontera, Temuco 4780000, Chile; 8 Carrera de Biotecnología, Facultad de Ciencias Agropecuarias y Medioambiente, Universidad de La Frontera, Temuco 4780000, Chile

**Keywords:** squamous skin cancer, photodynamic therapy, resistance, methyl aminolevulinate, ferrochelatase, clonogenic capability, porphobilinogen deaminase

## Abstract

Photodynamic therapy (PDT) treats nonmelanoma skin cancer. PDT kills cells through reactive oxygen species (ROS), generated by interaction among cellular O_2,_ photosensitizer and specific light. Protoporphyrin IX (PpIX) is a photosensitizer produced from methyl aminolevulinate (MAL) by heme group synthesis (HGS) pathway. In PDT-resistant cells, PDT efficacy has been improved by addition of epigallocatechin gallate (EGCG). Therefore, the aim of this work is to evaluate the effect of EGCG properties over MAL-TFD and PpIX production on A-431 cell line. EGCG’s role over cell proliferation (flow cytometry and wound healing assay) and clonogenic capability (clonogenic assay) was evaluated in A-431 cell line, while the effect of EGCG over MAL-PDT was determined by cell viability assay (MTT), PpIX and ROS detection (flow cytometry), intracellular iron quantification and gene expression of HGS enzymes (RT-qPCR). Low concentrations of EGCG (<50 µM) did not have an antiproliferative effect over A-431 cells; however, EGCG inhibited clonogenic cell capability. Furthermore, EGCG (<50 µM) improved MAL-PDT cytotoxicity, increasing PpIX and ROS levels, exerting a positive influence on PpIX synthesis, decreasing intracellular iron concentration and modifying HGS enzyme gene expression such as *PGB* (upregulated) and *FECH* (downregulated). EGCG inhibits clonogenic capability and modulates PpIX synthesis, enhancing PDT efficacy in resistant cells.

## 1. Introduction

Skin cancer has two main types, melanoma and nonmelanoma (NMSC), which are the most prevalent neoplasms worldwide. Melanoma represents 20% of cases and NMSC the remaining 80%, affecting approximately 2–3 million people [1]. In the particular case of NMSC, it is subdivided into basal cell carcinoma (BCC) and squamous cell carcinoma (SCC) or epidermoid, constituting 80% and 20% of cases, respectively. Some preneoplastic lesions, such as actinic keratosis [2] and Bowen’s disease [3], can cause SCC. In the case of NMSC, surgery is considered the standard therapy for its treatment [4]; however, not all lesions can be removed surgically due to their size, location or the patient’s condition. Photodynamic therapy (PDT) becomes a therapeutic alternative, since it is a treatment, approved by the Food and Drug Administration (FDA), that can be used on NMSC lesions with a depth of less than or equal to 2 mm [5]. PDT offers advantages over surgery, with excellent aesthetic and therapeutic results [6]. Additionally, it is a noninvasive technique that does not require an anesthetic procedure. It can be applied to large areas of skin or lesions that are difficult to access, particularly providing satisfactory cosmetic results. It is worth mentioning that PDT is not authorized by the FDA for the treatment of melanoma skin cancer due to the resistance of this type of cancer, caused by melanin competing with the photosensitizer for photon absorption [7], unlike NMSC [8].

In dermatology, to perform PDT, a cream containing a photosensitizer (PS) is applied to the lesion area and subsequently irradiated with visible light; the interaction of light, PS and cellular O_2_ generates toxic reactive oxygen species (ROS). Methyl-aminolevulinate (MAL) [9] is a precursor of the PS protoporphyrin IX (PpIX). PpIX is synthesized in the cells through the heme biosynthesis pathway from MAL. This mechanism favors the accumulation of PpIX within cellular compartments [10], mainly in the mitochondria, where it generates cytotoxicity through ROS. Among the limitations that have been found in PDT is the recurrence of lesions [11] associated to factors of resistance such as amount of cellular O_2_, the amount of PS absorbed by the cell and its level of accumulation, and the participation of molecules with antioxidant activity for the neutralization of ROS [12].

In the search to increase the effectiveness of PDT, epigallocatechin gallate (EGCG) is found, a polyphenol characteristic of green tea [13], which has antioxidant and pro-oxidant properties, depending on the concentrations and applications in which it is used [14]. The ability of EGCG to generate reactive oxygen species (ROS) [15] and influence cell signaling pathways [15] related to cell growth and apoptosis makes it a promising complement to PDT. In addition to these activities in oxidation, EGCG acts as a chelator [16], decreasing the availability of iron necessary for heme synthesis, which could increase the intracellular accumulation of PS and the effectiveness of PDT.

It has been described that EGCG improves the cytotoxic effect of PDT [17,18,19]; it has been observed to have effects in lymphoma through a mechanism of inhibition of cell proliferation, DNA fragmentation, and induction of apoptosis [17], in lung cancer, action through increasing levels of p21 and p53, as well as significantly expressed Bax and activated PARP genes in the combination [18]. In the case of breast cancer, the inclusion of EGCG in PDT increases tumor apoptosis and decreases the expression of pro-survival and angiogenic molecules within the tumor microenvironment [19]. Particularly in resistant in vitro models of NMSC, EGCG, through the increase in PpIX and the production of ROS, improves cytotoxicity compared to conventional PDT (MAL and red light). It was further shown that it allowed the regulation of gene expression associated with heme metabolism. Finally, chemoproteomic analyses identified numerous direct targets of EGCG within cellular compartments [20].

This research aims to address the gaps in knowledge regarding resistance to photodynamic therapy (PDT) and determine the mechanism through which EGCG enhances the effects of conventional PDT applied in the treatment of nonmelanoma skin cancer. Therefore, the in vitro effect of EGCG on the heme synthesis pathway and the production of protoporphyrin IX was studied to elucidate the mechanisms underlying the interaction of EGCG with PDT to develop therapeutic approaches more effective against skin cancer, especially in cases resistant to conventional treatments.

## 2. Results

### 2.1. EGCG at Low Concentrations Does Not Have an Antiproliferative Effect over A-431 Cells; However, It Inhibits Their Clonogenic Capability

The EC_50_ concentration of EGCG was determined by MTT assay at 4 h post treatment, using EGCG concentrations of 10–100 µM. The resulting EC_50_ was 54 µM at 4 h of incubation. Subsequently, the analysis of cell viability, clonogenic capability, wound healing assay and flow cytometry evaluation of proliferation were carried out with lower EGCG concentrations (10 µM, 20 µM and 40 µM). Due to incubation with MAL, a precursor of the photosensitizer PpIX, carried out for 4 h, all assays with EGCG were carried out during that time.

**Figure 1:**
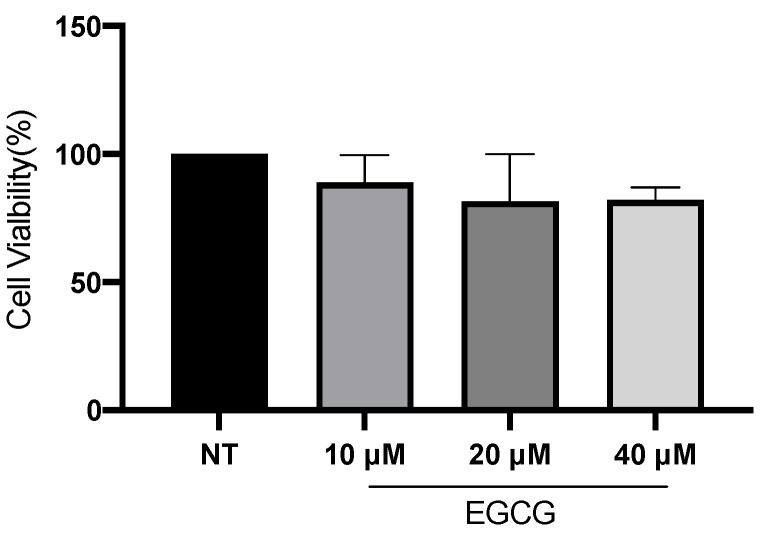
**A-431 cell viability evaluated by MTT assay 24 h after EGCG treatments**. Each experimental group was compared with its respective control (NT). Values of *p* < 0.05 were considered statistically significant. Data are expressed as mean ± SD of three biological replicates.

**Figure 2:**
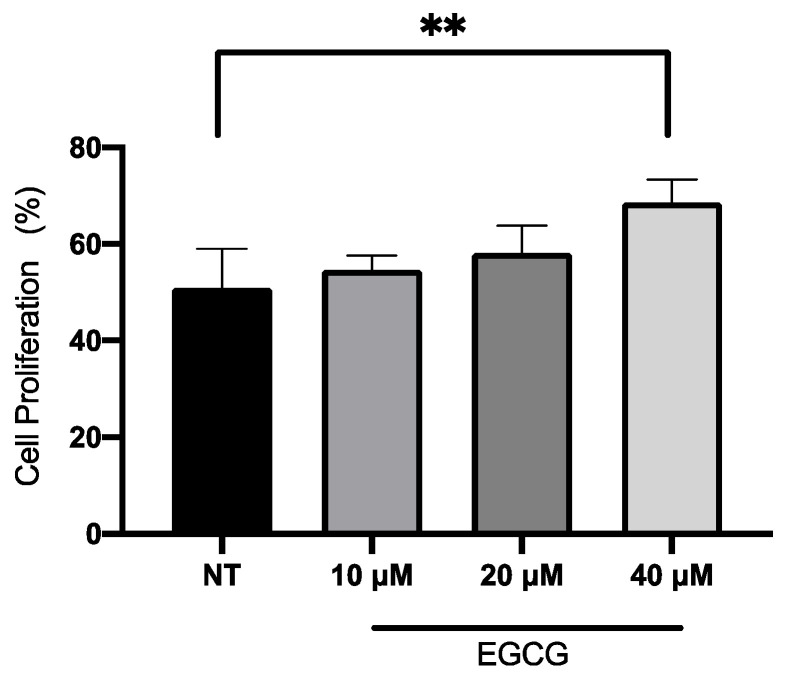
**Proliferation assay of A-431 cells incubated with 10 µM, 20 µM and 40 µM EGCG for 4 h.** Each experimental group was compared with its respective control (NT). Values of *p* < 0.05 were considered statistically significant. ** *p* < 0.01. Data are expressed as mean ± SD of three biological replicates.

In relation to A-431 cell viability, it was measured by MTT assay 24 h after EGCG treatment. As shown in Figure 1, EGCG at different concentrations did not reduce cell viability. A-431 cells maintained a viability percentage of more than 80%. Regarding the effect of EGCG on cell proliferation, cells were treated with EGCG 10 µM, 20 µM and 40 µM for 4 h and DNA synthesis was evaluated by flow cytometry. It was found that EGCG at 10 µM and 20 µM did not significantly affect the proliferation of A-431 cells, as shown in Figure 2. However, EGCG at 40 µM significantly increased cell proliferation percentage.

**Figure 3:**
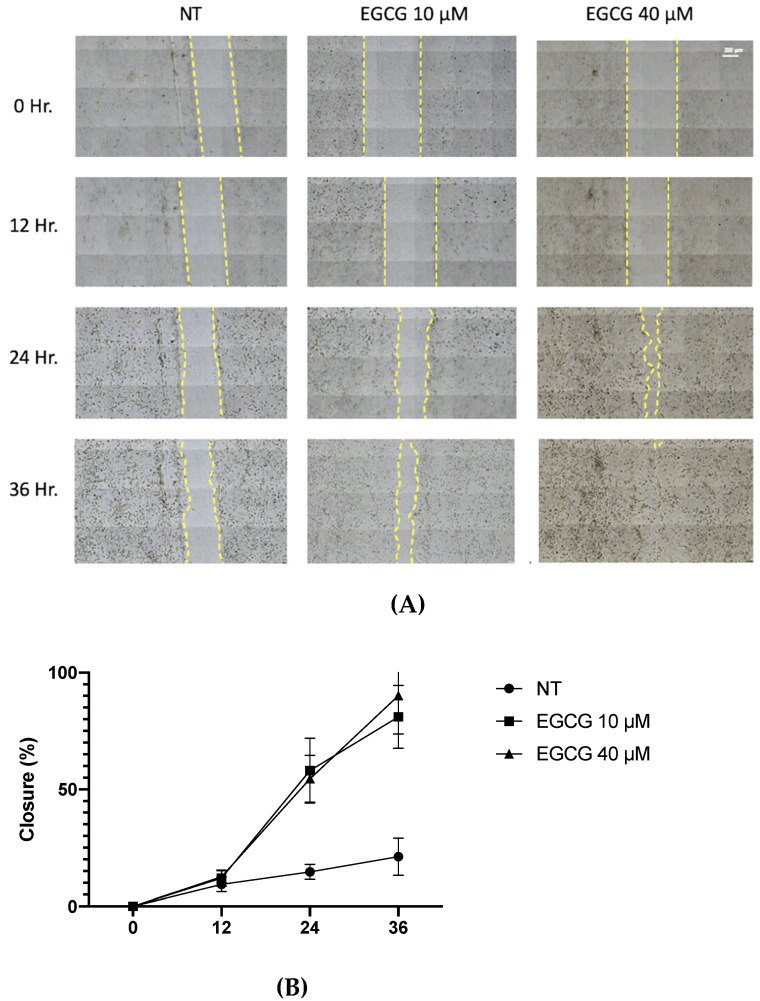
**Wound healing assay of A-431 cells incubated with 10 µM and 40 µM EGCG.** (**A**) Representative images of wound healing assay using an objective with 20× magnification. (**B**) Percentage of wound closure. Each experimental group was compared with its respective control (NT). Values of *p* < 0.05 were considered statistically significant. Data are expressed as mean ± SD of three biological replicates.

**Figure 4:**
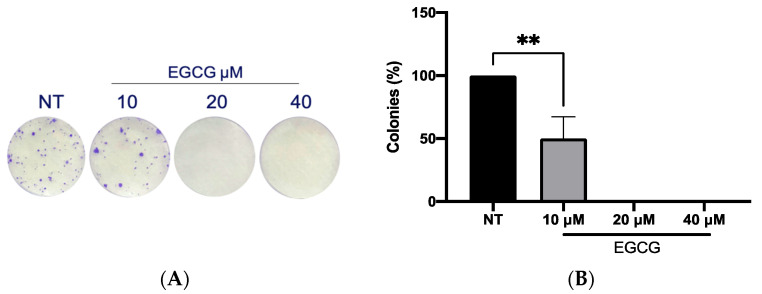
**Clonogenic assay of A-431 cells incubated with 10 µM, 20 µM and 40 µM EGCG.** (**A**) Colony formation. (**B**) Percentage of colonies. Each experimental group was compared with its respective control (NT). Values of *p* < 0.05 were considered statistically significant. ** *p* < 0.01. Data are expressed as mean ± SD of three biological replicates.

On the other hand, a wound healing analysis was carried out with 10 µM and 40 µM EGCG. Three areas per well were photographed every 12 h for a total of 36 h. As shown in Figure 3, EGCG did not affect proliferation, with even a trend of faster closure being seen compared to the untreated control at EGCG 40 µM. Therefore, EGCG does not have a cytotoxic and an antiproliferative effect at low concentrations over A-431 cells with an incubation of 4 h. In spite of this, EGCG at concentrations of 10 µM and 40 µM was capable of inhibiting the clonogenic capability of A-431 cells, decreasing the number of colonies until no colonies are formed (Figure 4).

### 2.2. Low EGCG Concentrations Improve PDT Cytotoxicity, Increasing PpIX and ROS Levels in A-431 Cells

**Figure 5:**
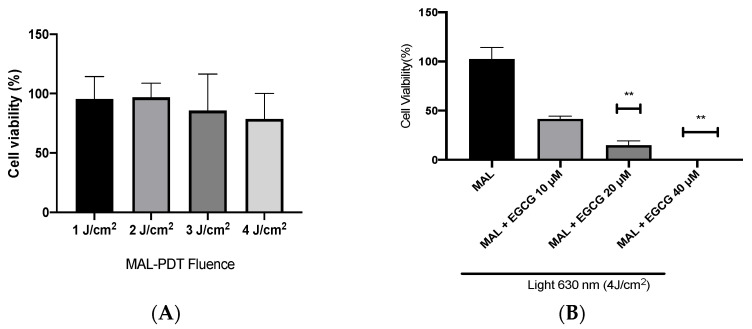
**A-431 cell viability evaluated by MTT assay 24 h after treatments.** (**A**) Intrinsic resistance to MAL-PDT of A-431 cells. (**B**) Effect of MAL-PDT + EGCG combination. Values of *p* < 0.05 were considered statistically significant. ** *p* < 0.01. Data are expressed as mean ± SD of three biological replicates.

**Figure 6:**
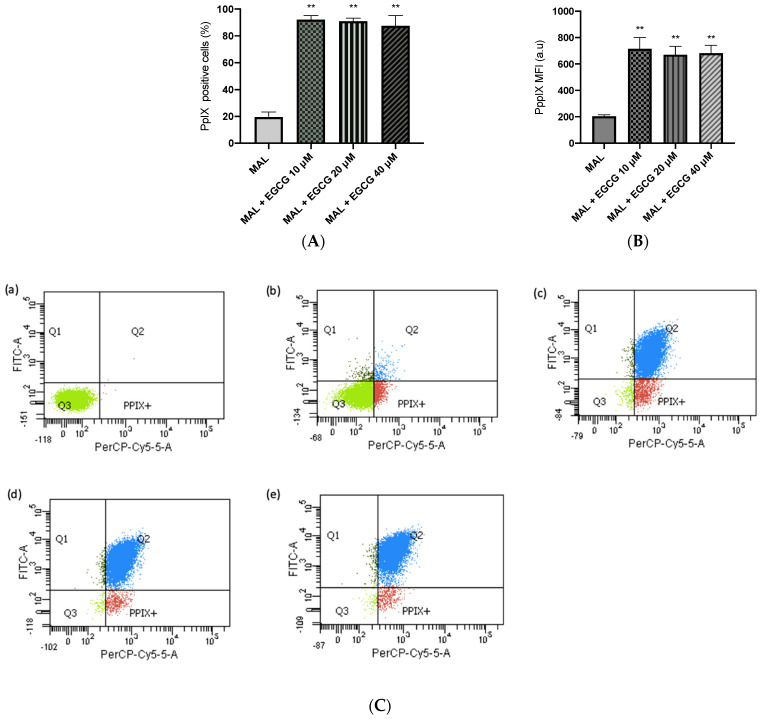
**PpIX levels in A-431 cells detected by flow cytometry**. (**A**) Cells positive for PpIX production. (**B**) Average fluorescence intensity of PpIX. (**C**) Representative flow cytometry charts: (**a**) autofluorescence control, (**b**) MAL–PDT, (**c**) MAL–PDT–EGCG 10 µM, (**d**) MAL–PDT–EGCG 20 µM and (**e**) MAL–PDT–EGCG 40 µM. Quadrants: Q3 (PpIX and ROS negative cells), Q4: PpIX positive cells, Q1: ROS positive cells, Q2 (PpIX and ROS positive cells). Values of *p* < 0.05 were considered statistically significant. ** *p* < 0.01. Data express mean ± SD of three biological replicates. a.u. = arbitrary units.

**Figure 7:**
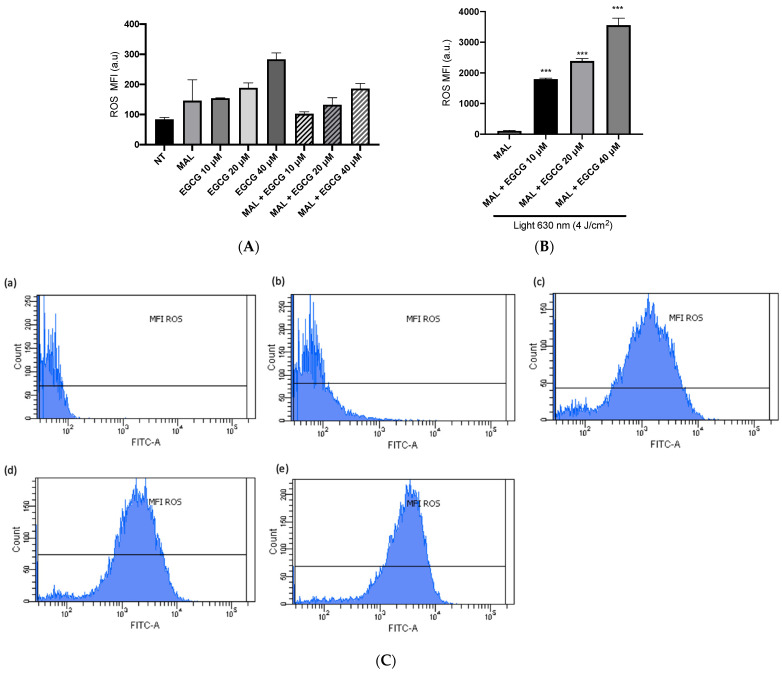
**ROS levels in A-431 cells detected by flow cytometry.** (**A**) Mean fluorescence intensity (MFI) of ROS in light-free controls of A-431 cells. (**B**) MFI of ROS in A-431 cells treated with MAL 2 mM and EGCG (10, 20 or 40 µM), with red light (630 nm, 4 J/cm^2^). (**C**) Representative histogram graphs: (**a**) autofluorescence control, (**b**) MAL-PDT, (**c**) MAL-PDT-EGCG 10 µM, (**d**) MAL-PDT-EGCG 20 µM and (**e**) MAL-PDT-EGCG 40 µM. Values of *p* < 0.05 were considered statistically significant. *** *p* < 0.001. Data are expressed as mean ± SD of three biological replicates. a.u. = arbitrary units.

A-431 cells present an intrinsic resistance to MAL-PDT; they are capable of maintaining over 90% of cells alive when treated with doses of 1–4 J/cm^2^ (Figure 5A). However, when combining EGCG with MAL-PDT, A-431 cell viability decreased to 40%, 15% and 0%, using MAL-PDT with EGCG 10 µM, 20 µM and 40 µM, respectively (Figure 5B). This greater cytotoxic effect of MAL-PDT associated with EGCG is also related to a significant increase in intracellular PpIX levels and total ROS generation. As shown in Figure 6A,C, only 20% of A-431 cells contain PpIX when treated with MAL; however, when EGCG is added in different concentrations, the cells containing PpIX increase to almost 100%. The above is also related to a greater fluorescence intensity of PpIX (Figure 6B). On the other hand, Figure 7A shows the total ROS generated by the cells when treated with MAL, EGCG and MAL + EGCG in the absence of light. When comparing these treatments with the NT control, there are no significant differences. However, when red light is applied, ROS levels increase significantly in A-431 cells treated with MAL-PDT + EGCG (Figure 6C and Figure 7B,C).

### 2.3. EGCG Exerts Positive Influence on PpIX Synthesis

**Figure 8:**
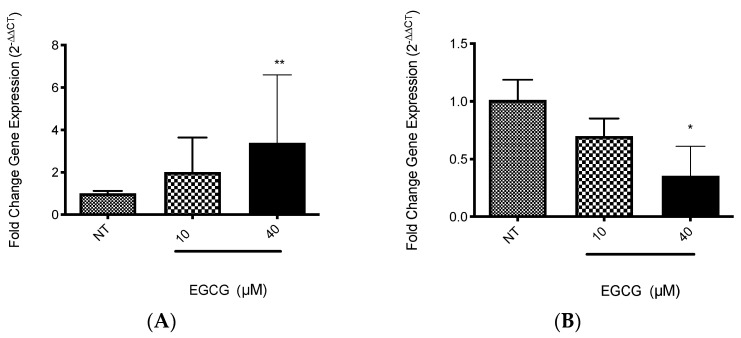
**Gene expression of relevant heme synthesis pathway enzymes in A-431 cells exposed to EGCG for 4 h.** (**A**) PGB gene. (**B**) FECH gene. Values of * *p* < 0.05 were considered statistically significant. ** *p* < 0.01. Data are expressed as mean ± SD of three biological replicates.

**Figure 9:**
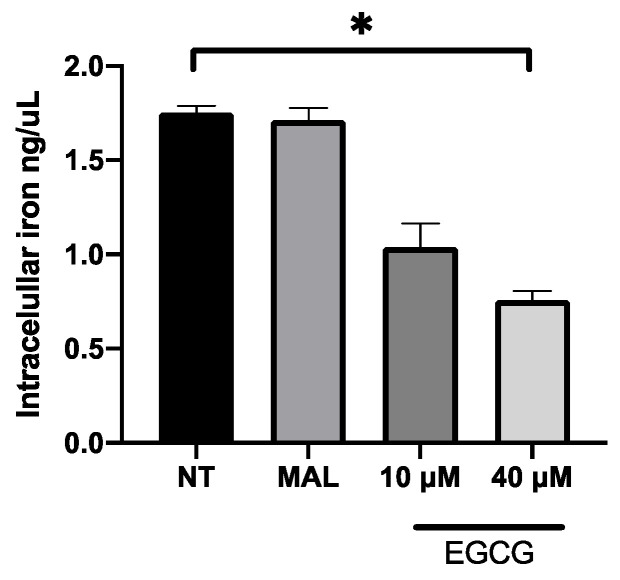
**Concentration of intracellular iron in A-431 cells exposed to EGCG for 4 h.** Values of *p* < 0.05 were considered statistically significant. * *p* < 0.05. Data are expressed as mean ± SD of three biological replicates.

EGCG was associated with the increase in PDT cytotoxicity in A-431 cells. The main finding was related with higher levels of intracellular PpIX in A-431 cells treated with MAL and EGCG. Therefore, gene expression of heme group synthesis enzymes was evaluated. A significant upregulation of the PGB enzyme gene was observed, which allows the synthesis of PpIX (Figure 8A). Meanwhile, there was a decrease in expression of the ferrochelatase enzyme transcript (FECH), which is responsible for heme group formation, due to the addition of an iron molecule to the PpIX ring (Figure 8B). In addition, EGCG treatment reduced the intracellular iron concentration of iron in A-431 cells, as shown in Figure 9.

## 3. Discussion

### 3.1. Effect of EGCG on A-431 Cell Proliferation and ROS Generation

Epigallocatechin gallate (EGCG) is the most abundant component of green tea [13]. The cytotoxic effect of EGCG is mediated by ROS and it promotes apoptosis through mitochondrial damage, membrane depolarization and release of cytochrome C, which is inhibited by catalase or other antioxidant molecules [21]. EGCG is capable of inducing autophagy, which depends on EGCG concentration, stress conditions and cell type [22]. According to concentration, high concentrations of EGCG (100 μM) inhibit autophagy, leading to apoptosis. In contrast, low concentrations of EGCG (10 μM) induce autophagy [23]. While in studies with animal models of carcinogenesis, EGCG and green tea extracts were capable of inhibiting tumorigenesis during the initiation, promotion and progression stages [24], in this work, the antiproliferative role of EGCG was analyzed. First of all, A-431 cells exposed to EGCG at low concentrations (10 µM, 20 µM and 40 µM) for 4 h did not reduce their metabolic viability (MTT assay). Furthermore, cell proliferation was determined by flow cytometry as active DNA synthesis in the cell. It was found that EGCG at 10 µM and 20 µM did not significantly affect the cell proliferation. In contrast, EGCG at 40 µM significantly increased cell proliferation percentage. Furthermore, wound healing assay was used to evaluate cell proliferation because, in the presence of medium + FBS and a realization time greater than 112 h, without DNA inhibitors, it provides valuable information about the cell multiplication process [25,26]. Consequently, wound closure would be an effect primarily dependent on proliferation. In fact, wound closure occurs faster in cells treated with EGCG after 36 h of incubation. Most research mentions the potent effect of EGCH in inhibiting cell proliferation and inducing apoptosis [27,28]. While some works characterize the promoting action on cell proliferation. In HaCat cells, EGCG (<12.5 μM, >24 h) shows antioxidant and cytoprotective capabilities [29]. Also, in other keratinocytes, EGCG (<200 μM) [30] induces differentiation and proliferation, which would help in skin wound healing [30,31]. In addition, in this study, an interesting finding was related to clonogenic capability. In comparison with proliferation, EGCG (<50 μM) was capable of inhibiting colony growth. Therefore, in this cell model, EGCG in low concentrations induces proliferation and inhibits clonogenic capability.

On the other hand, EGCG could have an antioxidant and pro-oxidant role because its structure is rich in aromatic rings and hydroxyl groups [14]. Its structure is unstable, which favors the processes of auto-oxidation (20–100 µM) and epimerization (>mM) and it can lead to degradation [15,24]. In this study, it was evaluated that ROS generation is associated only to EGCG by measuring total ROS (flow cytometry). It was observed that the presence of EGCG at low concentrations (<50 µM) did not produce significant levels of ROS on A-431 cells; even so, a slight tendency of increasing ROS intensity (a.u.) was observed. Therefore, it is not possible to assign a pro-oxidant role. Other in vitro studies indicate that EGCG acts as a pro-oxidant agent by producing H_2_O_2_ and, also, generating more potent ROS as hydroxyl radicals (-OH) directly promoting cytotoxicity [21,32]. The combination between H_2_O_2_ and these radicals has been observed in high concentrations of EGCG (>50 μM) [21]. Therefore, the concentrations used in this work would not be sufficient to generate the EGCG pro-oxidant effect.

### 3.2. EGCG Positively Influences PpIX Synthesis and Improves PDT for Resistant Cells

Briefly, PDT consists of three components: O_2_, photosensitizer and light [33]. Methyl aminolevulinate (MAL) is metabolized by cells by the heme synthesis pathway and it accumulates in mitochondria as protoporphyrin IX (PpIX) [9], a PDT photosensitizer. PpIX is excited by red light and reacts with cellular O_2_ to generate reactive oxygen species (ROS). Finally, ROS cause cytotoxicity and cell death [34,35]. Additionally, some reports show EGCG improves the effect of PDT, both in in vivo and in vitro models, decreasing the viability of human Jurkat cells (leukemia) [17], TC-1 cells (lung tumor of mouse) [18] and BA cells (mouse breast cancer) [19]. Previous findings demonstrated that NMSC-resistant cells to MAL-PDT had lower levels of intracellular PpIX and ROS than sensitive cells. Nevertheless, the addition of epigallocatechin gallate (EGCG) to MAL-PDT improves cytotoxicity [36]. The current study corroborated this cytotoxic effect in A-431 cells, where PpIX and ROS levels also increased. Trying to understand the EGCG mechanism in A-431 cells exposed to MAL-PDT, intracellular iron quantification and analysis of RT-qPCR targeted to the heme synthesis pathway were performed. In our results, EGCG reduces available iron through its chelating action, which correlates with the generation of ROS mentioned earlier and influences the PPIX formation pathway. The chelation of iron by EGCG decreases its availability, which, in conjunction with PpIX, forms the heme group catalyzed by the enzyme ferrochelatase (FECH), leading to increased accumulation of PpIX within the cell [36]. Due to characteristic EGCG structure [37], EGCG is able to chelate metal ions, leading to its autoxidation and generation of hydrogen peroxide, which induces cell apoptosis [38]. Chelation of transition metal ions can interfere with the delivery of metal ion cofactors to receptor kinases [39] and regulate enzymes [40]. Among the main ions chelated are cadmium [41], copper [42] and iron [43]. Iron is crucial for normal cell function; however, in tumor cells, excess iron promotes progression [44]. Conversely, chelation of iron has been utilized to promote apoptosis in colon cancer cases, using desferrioxamine [44]. Specifically, EGCG has been observed to decrease iron levels through its chelation activity in HT-29 colorectal cancer cells, inducing endoplasmic reticulum stress and favoring apoptosis [45]. This property is important in PDT, reducing the availability of iron for synthesis of the heme group; it allows a greater accumulation of intracellular PpIX.

Regarding the FECH enzyme, which is the final enzyme in the heme biosynthesis pathway, EGCG decreases its expression in our experimental model. FECH catalyzes the insertion of ferrous iron into PpIX to form heme, an important protein incorporated into the electron transport chain that regulates ATP production. In various cancers, including bladder cancer, inactive FECH results in reduced heme production and downregulation of PpIX catabolism, thereby favoring PpIX accumulation [46]. In an in vitro model using prostate cancer cells (PC-3) treated with 5-aminolevulinic acid (ALA) and PDT, the efficiency was enhanced by using an FECH inhibitor. Inhibiting FECH with deferoxamine and NOC-18 increased PpIX accumulation [47]. Similarly, in an in vitro model of human urothelial cancer, adding deferoxamine, an FECH inhibitor, increased PpIX accumulation and improved the effect of ALA-PDT [48]. In a colon cancer model with Caco-2 cells, loss of FECH was found to be protective against colon cancer tumorigenesis in vitro [49]. Additionally, our findings indicate that EGCG upregulates the PGB enzyme responsible for forming the PpIX ring. This suggests that EGCG enhances the effect of MAL by increasing PpIX accumulation through metal chelation, reducing enzyme expression and overexpressing PGB.

In conclusion, EGCG has a positive effect on MAL-PDT; the molecular findings show part of the mechanism that could be involved in EGCG participation. Even so, although more research is needed on this process, these results show the great benefit of the combination of this polyphenol with MAL-PDT in the treatment of nonmelanoma skin cancer.

## 4. Materials and Methods

### 4.1. Cell Culture

Human skin cell line A-431, derived from squamous cell carcinoma, was kindly donated by Dr. Manuel Grez, Georg-Speyer-Haus Institute for Tumor Biology and Experimental Therapy, Germany. The A-431 cell line was used because it shows intrinsic resistance to MAL-PDT under conditions of 2 mM MAL and 4 J/cm^2^. Thus, it is a resistance model that allows the evaluation of the effect of EGCG on MAL-PDT.

A-431 cells were cultured in Dulbecco’s minimal essential medium (DMEM) high glucose (HyClone), supplemented with 10% fetal bovine serum (FBS) and 1% penicillin/streptomycin. Cells were maintained in a humidified incubator at 37 °C with 5% CO_2_.

### 4.2. Reagents Preparation

Stock solution of methyl aminolevulinate (#CS-W009212, Chemscene, Monmouth Junction, NJ, USA) was prepared in PBS 1X at 1 M; meanwhile, work solution was prepared at 2 mM in serum-free DMEM medium without phenol red. Epigallocatechin gallate (#CS-1258, Chemscene) was dissolved in PBS 1X, preparing a stock solution of 10 mM and using a work solution (10, 20 and 40 µM) in DMEM medium without phenol red. Depending on the assay, FBS was used or not. Stock solutions were stored in amber tubes at −20 °C. To prevent degradation of EGCG and MAL compounds, work solutions were prepared on the same day of the assay, and the stock solution used was eliminated.

### 4.3. MTT Assay

Cell viability was evaluated by MTT assay 24 h after treatments (EGCG and MAL-PDT with or without EGCG). MTT (Sigma-Aldrich, St. Louis, MO, USA) solution was prepared in serum-free DMEM medium without phenol red at a final concentration of 0.1 mg/mL. Medium was discarded and cells were washed once with Dulbecco’s phosphate-buffered saline (DPBS) 1X (HyClone, Washington, DC, USA). MTT was added and cells were incubated for 2 h in darkness at 37 °C with 5% CO_2_. Then, medium with MTT was removed and formazan crystals were dissolved with isopropanol. Absorbance was measured at 570 nm with a background subtraction at 690 nm using a multi-well plate reader.

### 4.4. EC_50_ of EGCG Assay

Briefly, cells were seeded in 96-well plates at a concentration of 2000 cells/well. After 24 h, the cells were washed and were incubated with DMEM without phenol red or with EGCG diluted in the same medium in different concentrations (0–100 µM). They were allowed to incubate for 4 h in darkness at 37 °C with 5% CO_2_. Then, cells were washed and fresh complete DMEM was added. The next day, viability was evaluated by MTT.

### 4.5. Proliferation Assay

Cells were seeded in 6-well plates, 1 × 10^6^ cells/well. After 24 h, the cells were washed twice with DPBS 1X and starved for 24 h in serum-free DMEM. Then, cells were incubated with EGCG 10, 20 and 40 µM for 4 h in darkness at 37 °C with 5% CO_2_. The Click-iT TM Plus EdU Flow Cytometry Assay Kit (C10632, Invitrogen, Waltham, MA, USA) was used to detect DNA synthesis by flow cytometry (BD FACSCanto™ II). This kit was used according to the instructions of the manufacturer.

### 4.6. Wound Healing Assay

Cells were seeded in 24-well plates, at a total of 1 × 10^5^ cells/well. After 24 h, upon reaching 90% confluence (ideally 100%), the wound was made using a P200 micropipette tip. Immediately, cells were washed twice with DPBS to remove any debris or cells that remained in suspension. Then, cells were incubated with EGCG 10, 20 and 40 µM dissolved in DMEM with FBS and without phenol red in darkness at 37 °C with 5% CO_2_. Control cells were incubated with the same DMEM medium. Three areas per well were photographed (using an objective with 20× magnification), every 12 h, for a total of 36 h, using TissueFAXS i Plus Cytometer Microscopy (TissueGnostics, Vienna, Austria).

### 4.7. Clonogenic Assay

A concentration of 500 cells/well were seeded in 6-well plates. After 24 h, cells were incubated with EGCG 10, 20 and 40 µM prepared in DMEM with FBS and without phenol red for 4 h in darkness at 37 °C with 5% CO_2_. Colonies were observed after 14 days of incubation. Then, cells were washed with DPBS 1X and fixed with cold methanol. Colonies were stained with crystal violet.

### 4.8. In Vitro MAL-PDT

In vitro, MAL-PDT was carried out as mentioned by Leon et al., 2020 [36]. Briefly, cells were seeded in 24-well plates (1 × 10^5^ cells/well). After 24 h, cells were washed once with DPBS and incubated with MAL 2 mM. Then, cells were incubated in darkness for 4 h in a humidified incubator. Immediately, cells were irradiated with red light (630 nm) and 30 mW, using a light-emitting diode (LED) device. After irradiation, medium was removed and replaced with complete culture medium and cells maintained in a humidified incubator. In the case of MAL-PDT + EGCG, a solution of MAL and EGCG (10–40 µM) was prepared to treat cells at the same time (incubation of 4 h).

### 4.9. PpIX and Total ROS Detection

Both, PpIX and total ROS detection were previously standardized [36]. PpIX shows intrinsic fluorescence; therefore, it could be detected by flow cytometry. Cells were seeded in 24-well plates (10^5^ cells/well) and cultured for 24 h. Next, cells were incubated with a 2 mM solution of MAL with or without EGCG (10, 20 and 40 µM) for 4 h in darkness at 37 °C with 5% CO_2_. Cells were detached by trypsinization and centrifuged and washed. The pellet was resuspended in 200 µL of DPBS 1X to be analyzed by a flow cytometer (λ_Ex_: 405 nm, λ_Em_: 633 nm). Technical controls: autofluorescence (cells without treatment).

Total ROS detection was performed using the nonfluorescent probe CM-H_2_DCFDA for flow cytometry. Cells were treated with MAL-PDT (MAL 2 mM) and MAL-PDT + EGCG (10, 20 and 40 µM), both with red light irradiation using 4 J/cm^2^. Before red light irradiation, cells were washed once and incubated with CM-H_2_DCFDA 1 µM for 40 min in darkness at 37 °C. Then, medium was discarded and replaced with fresh complete medium to be incubated at 37 °C for 10 min. Cells were trypsinized, centrifuged and the pellet was washed with 1 mL of DPBS 1X. Then, cells were analyzed by flow cytometer using λ_Ex_ 492–495 nm and λ_Em_ 517–527 nm. Technical controls: autofluorescence (cells without ROS probe and treatment), basal ROS (cells with ROS probe and without treatment) and positive control (cells incubated with H_2_O_2_ and ROS probe).

Both assays were analyzed by a flow cytometer (BD FACSCanto™ II) reading 1 × 10^4^ events.

### 4.10. Gene Expression Analysis

Cells were seeded in T25 plates. When confluence was 80%, cells were exposed to different treatments: EGCG 10 µM and EGCG 40 µM and untreated control (medium) for 4 h at 37 °C in darkness. As a control, cells were incubated with DMEM without phenol red and the same medium was used to prepare treatment solutions. After 4 h of treatment, RNA extraction was performed using TRIzol^®^ reagent and cDNA was prepared from RNA 1 µg. RT-qPCR was used to evaluate the differential gene expression of heme synthesis pathway enzymes, *PGB* and *FECH*, based on the protocol and primer sequences mentioned by Leon et al., 2020 [36]. *GAPDH* and *ACTB* were used as housekeeping and relative mRNA expression was determined using a 2^−ΔΔCT^ method [50].

### 4.11. Iron Quantification Assay

Cells were seeded in T25 flasks. After 24 h, cells were treated with MAL (2 mM) or EGCG (10 or 40 µM) and incubated for 4 h at 37 °C in darkness. Then, cells were washed and the quantification was performed using Iron Assay Kit (#MAK025, Sigma-Aldrich) according to the instructions of the manufacturer.

### 4.12. Statistical Analysis

Data were presented as mean ± SD and significance was analyzed with Mann Whitney test, using GraphPad Prism (GraphPad Software version 8, La Jolla, CA, USA). All assays were performed using technical and biological triplicates. Statistical significance was established at the *p* < 0.05 level.

## 5. Patents

These results are protected by patent application to the World Intellectual Property Organization (WIPO), PCT/IB2019/054042, reference number 2019-17309.

## Data Availability

The original contributions presented in this study are included in the article; further inquiries can be directed to the corresponding authors.

## References

[1] World Health Organization (2013). Ultraviolet Radiation and the INTERSUN Programme.

[2] Gupta A., Paquet M., Villanueva E., Brintnell W. (2012). Interventions for Actinic Keratoses (Review). Cochrane Database Syst. Rev..

[3] Bath-Hextall F., Matin R., Wilkinson D., Leonardi-Bee J. (2013). Interventions for Cutaneous Bowen’ s Disease. Cochrane Database Syst. Rev..

[4] Berman B., Amini S. (2012). Pharmacotherapy of Actinic Keratosis: An Update. Expert Opin. Pharmacother..

[5] Morton C.A., Szeimies R.-M., Sidoroff A., Braathen L.R. (2013). European Guidelines for Topical Photodynamic Therapy Part 1: Treatment Delivery and Current Indications–Actinic Keratoses, Bowen’s Disease, Basal Cell Carcinoma. J. Eur. Acad. Dermatol. Venereol..

[6] Li Q., Gao T., Jiao B., Hu X., Luan Q., Li K., Ma C., Li C., Wang S. (2011). Tumor Thickness Predicts Long-Term Complete Response of Facial Basal Cell Carcinomas in Asian Skin Types Iv/v Treated with Methyl Aminolaevulinate Photodynamic Therapy. Photomed. Laser Surg..

[7] Huang Y.-Y., Vecchio D., Avci P., Yin R., Garcia-Diaz M., Hamblin M.R. (2013). Melanoma Resistance to Photodynamic Therapy: New Insights. Biol. Chem..

[8] Lucena S.R., Salazar N., Gracia-Cazaña T., Zamarrón A., González S., Juarranz Á., Gilaberte Y. (2015). Combined Treatments with Photodynamic Therapy for Non-Melanoma Skin Cancer. Int. J. Mol. Sci..

[9] O’Connor A.E., Gallagher W.M., Byrne A.T. (2009). Porphyrin and Nonporphyrin Photosensitizers in Oncology: Preclinical and Clinical Advances in Photodynamic Therapy. Photochem. Photobiol..

[10] Fiorito V., Allocco A.L., Petrillo S., Gazzano E., Torretta S., Marchi S., Destefanis F., Pacelli C., Audrito V., Provero P. (2021). The Heme Synthesis-Export System Regulates the Tricarboxylic Acid Cycle Flux and Oxidative Phosphorylation. Cell Rep..

[11] Cavicchini S., Serini S.M., Fiorani R., Girgenti V., Ghislanzoni M., Sala F. (2011). Long-Term Follow-up of Metil Aminolevulinate (MAL)-PDT in Difficult-to-Treat Cutaneous Bowen’s Disease. Int. J. Dermatol..

[12] Casas A., Di Venosa G., Hasan T. (2011). Al Batlle Mechanisms of Resistance to Photodynamic Therapy. Curr. Med. Chem..

[13] Du G., Zhang Z., Wen X., Yu C., Calway T., Yuan C., Wang C. (2012). Epigallocatechin Gallate (EGCG) Is the Most Effective Cancer Chemopreventive Polyphenol in Green Tea. Nutrients.

[14] Shuang S., Ye-Wei H., Yang T., Xuan-Jun W., Jun S. (2014). Mechanism of Action of (−)-Epigallocatechin-3-Gallate: Auto-Oxidation-Dependent Activation of Extracellular Signal-Regulated Kinase 1/2 in Jurkat Cells. Chin. J. Nat. Med..

[15] Krupkova O., Ferguson S.J., Wuertz-kozak K. (2016). Stability of (−)-Epigallocatechin Gallate and Its Activity in Liquid Formulations and Delivery Systems. J. Nutr. Biochem..

[16] Min K., Kwon T.K. (2014). Anticancer Effects and Molecular Mechanisms of Epigallocatechin-3-Gallate. Integr. Med. Res..

[17] Qi H., Abe N., Zhu B., Murata Y., Nakamura Y. (2014). (−)-Epigallocatechin-3-Gallate Ameliorates Photodynamic Therapy Responses in an in Vitro T Lymphocyte Model. Phyther. Res..

[18] Mun S.T., Bae D.H., Ahn W.S. (2014). Epigallocatechin Gallate with Photodynamic Therapy Enhances Anti-Tumor Effects In Vivo and In Vitro. Photodiagn. Photodyn. Ther..

[19] Ferrario A., Luna M., Rucker N., Wong S., Gomer C.J. (2011). Pro-Apoptotic and Anti-Inflammatory Propierties of the Green Tea Constituent Epigallocatechin Gallate Increase Photodynamyc Therapy Resposiveness. Lasers Surg. Med..

[20] Sahadevan R., Binoy A., Vechalapu S., Nanjan P., Sadhukhan S. (2023). In Situ Global Proteomics Profiling of EGCG Targets Using a Cell- Permeable and Click-Able Bioorthogonal Probe. Int. J. Biol. Macromol..

[21] Kim H., Quon M.J., Kim J. (2014). New Insights into the Mechanisms of Polyphenols beyond Antioxidant Properties; Lessons from the Green Tea Polyphenol, Epigallocatechin 3-Gallate. Redox Biol..

[22] Zhang Y., Yang N.D., Zhou F., Shen T., Duan T., Zhou J., Shi Y., Zhu X.Q., Shen H.M. (2012). (−)-Epigallocatechin-3-Gallate Induces Non-Apoptotic Cell Death in Human Cancer Cells via ROS-Mediated Lysosomal Membrane Permeabilization. PLoS ONE.

[23] Li W., Zhu S., Li J., Assa A., Jundoria A., Xu J., Fan S., Eissa N.T., Tracey K.J., Sama A.E. (2011). EGCG Stimulates Autophagy and Reduces Cytoplasmic HMGB1 Levels in Endotoxin-Stimulated Macrophages. Biochem. Pharmacol..

[24] Lambert J.D., Elias R.J. (2010). The Antioxidant and Pro-Oxidant Activities of Green Tea Polyphenols: A Role in Cancer Prevention. Arch. Biochem. Biophys..

[25] Giretti M.S., Guevara M.M.M., Cecchi E., Mannella P., Palla G., Spina S., Bernacchi G., Di Bello S., Genazzani A.R., Genazzani A.D. (2014). Effects of Estetrol on Migration and Invasion in T47-D Breast Cancer Cells through the Actin Cytoskeleton. Front. Endocrinol..

[26] Latifi-Pupovci H., Kuçi Z., Wehner S., Bönig H., Lieberz R., Klingebiel T., Bader P., Kuçi S. (2015). In Vitro Migration and Proliferation (“wound Healing”) Potential of Mesenchymal Stromal Cells Generated from Human CD271+ Bone Marrow Mononuclear Cells. J. Transl. Med..

[27] Luo K.W., Chen W., Lung W.Y., Wei X.Y., Cheng B.H., Cai Z.M., Huang W.R. (2017). EGCG Inhibited Bladder Cancer SW780 Cell Proliferation and Migration Both In Vitro and In Vivo via Down-Regulation of NF-ΚB and MMP-9. J. Nutr. Biochem..

[28] Jiang S., Huang C., Zheng G., Yi W., Wu B., Tang J., Liu X., Huang B., Wu D., Yan T. (2022). EGCG Inhibits Proliferation and Induces Apoptosis through Downregulation of SIRT1 in Nasopharyngeal Carcinoma Cells. Front. Nutr..

[29] Kim E., Han S.Y., Hwang K., Kim D., Kim E.M., Hossain M.A., Kim J.H., Cho J.Y. (2019). Antioxidant and Cytoprotective Effects of (−)-Epigallocatechin-3-(3″-o-Methyl) Gallate. Int. J. Mol. Sci..

[30] Hsu S., Bollag W.B., Lewis J., Huang Q., Singh B., Sharawy M., Yamamoto T., Schuster G. (2003). Green Tea Polyphenols Induce Differentiation and Proliferation in Epidermal Keratinocytes. J. Pharmacol. Exp. Ther..

[31] Xu F.W., Lv Y.L., Zhong Y.F., Xue Y.N., Wang Y., Zhang L.Y., Hu X., Tan W.Q. (2021). Beneficial Effects of Green Tea EGCG on Skin Wound Healing: A Comprehensive Review. Molecules.

[32] Aggarwal V., Tuli H.S., Tania M., Srivastava S., Ritzer E.E., Pandey A., Aggarwal D., Barwal T.S., Jain A., Kaur G. (2020). Molecular Mechanisms of Action of Epigallocatechin Gallate in Cancer: Recent Trends and Advancement. Semin. Cancer Biol..

[33] Josefsen L.B., Boyle R.W. (2008). Photodynamic Therapy: Novel Third-Generation Photosensitizers One Step Closer?. Br. J. Pharmcol..

[34] Youle R., Strasser A. (2008). The BCL-2 Protein Family: Opposing Activities That Mediate Cell Death. Nat. Rev. Mol. Cell Biol..

[35] Shiozaki E., Shi Y. (2004). Caspases, IAPs and Smac/DIABLO: Mechanisms from Structural Biology. Trends Biochem. Sci..

[36] León D., Buchegger K., Silva R., Riquelme I., Viscarra T., Mora-Lagos B., Zanella L., Schafer F., Kurachi C., Roa J.C. (2020). Epigallocatechin Gallate Enhances MAL-PDT Cytotoxic Effect on PDT-Resistant Skin Cancer Squamous Cells. Int. J. Mol. Sci..

[37] Orisakwe O.E., Amadi C.N., Frazzoli C. (2020). Management of Iron Overload in Resource Poor Nations: A Systematic Review of Phlebotomy and Natural Chelators. J. Toxicol..

[38] Li D., Cao D., Cui Y., Sun Y. (2023). The Potential of Epigallocatechin Gallate in the Chemoprevention and Therapy of Hepatocellular Carcinoma. Front. Pharmacol..

[39] Mechanisms B. (2007). Reading the Tea Leaves: Anticarcinogenic Properties of (−)-Epigallocatechin-3-Gallate. Mayo Clin. Proc..

[40] Botten D., Fugallo G., Fraternali F., Molteni C. (2015). Structural Properties of Green Tea Catechins. J. Phys. Chem. B.

[41] An Z., Qi Y., Huang D., Gu X., Tian Y., Li P., Li H., Zhang Y. (2014). EGCG Inhibits Cd^2+^-Induced Apoptosis through Scavenging ROS Rather than Chelating Cd^2+^ in HL-7702 Cells. Toxicol. Mech. Methods.

[42] Farhan M. (2022). Green Tea Catechins: Nature’ s Way of Preventing and Treating Cancer. Int. J. Mol. Sci..

[43] Alam M., Ali S., Ashraf G., Bilgrami A.L., Kumar D., Hassan I. (2022). Epigallocatechin 3-Gallate: From Green Tea to Cancer Therapeutics. Food Chem..

[44] Nesran Z.N., Shafie N.H., Tohid S.F., Norhaizan M.E., Ismail A. (2020). Iron Chelation Properties of Green Tea Epigallocatechin-3-Gallate (EGCG) in Colorectal Cancer Cells: Analysis on Tfr/Fth Regulations and Molecular Docking. Evid.-Based Complement. Altern. Med..

[45] Nesran M., Nameyra Z. (2018). Role of Epigallocatechin-3-Gallate from Green Tea in Iron Chelation and Endoplasmic Reticulum Stress Pathway in Colorectal Cancer Cells. Master’s Thesis.

[46] Inoue K., Fukuhara H., Yamamoto S., Karashima T., Kurabayashi A., Furihata M., Hanazaki K., Lai H.W. (2022). Current Status of Photodynamic Technology for Urothelial Cancer. Cancer Sci..

[47] Fukuhara H., Inoue K., Kurabayashi A., Shuin T. (2013). The Inhibition of Ferrochelatase Enhances 5-Aminolevulinic Acid-Based Photodynamic. Photodiagn. Photodyn. Ther..

[48] Inoue K., Fukuhara H., Kurabayashi A., Furihata M., Tsuda M., Nagakawa K. (2013). Photodynamic Therapy Involves an Antiangiogenic Mechanism and Is Enhanced by Ferrochelatase Inhibitor in Urothelial Carcinoma. Cancer Sci..

[49] Safi R., Mohsen-kanson T., Nemer G., Dekmak B., Rubeiz N., El-sabban M., Nassar D., Eid A., Abbas O., Kibbi A. (2020). Loss of Ferrochelatase Is Protective against Colon Cancer Cells: Ferrochelatase a Possible Regulator of the Long Noncoding RNA H19. J. Gastrointest. Oncol..

[50] Livak K.J., Schmittgen T.D. (2001). Analysis of Relative Gene Expression Data Using Real-Time Quantitative PCR and the 2(-Delta Delta C(T)) Method. Methods.

